# Processes Associated with the Development of Depression in Autistic Individuals: A Narrative Review

**DOI:** 10.3390/healthcare13233112

**Published:** 2025-11-28

**Authors:** Yaerin H. Wallenberger, Mirko Uljarevic, Lacey Chetcuti, Samuel P. Putnam

**Affiliations:** 1 Department of Psychology, Bowdoin College, Brunswick, ME 04011, USA; 2 School of Medicine, Stanford University, Stanford, CA 94305, USA; 3 Yale Child Study Center, School of Medicine, Yale University, New Haven, CT 06520, USA

**Keywords:** autism, depression, mental health, mechanisms, social determinants, sensory sensitivity, internalized stigma, minority stress model, autistic camouflaging, emotion regulation, social communication

## Abstract

Autistic individuals are disproportionately likely to experience mental health challenges during their lifetime, with depressive disorders being particularly common. High rates of depression among autistic individuals are a particular matter of clinical and policy concern, given the well-established links between depression and a range of negative outcomes, including suicidality. By proposing a novel integrative model, we aim to synthesize existing knowledge and prompt innovation in previously under-explored areas. In this narrative review, we first briefly discuss the prevalence of depression in autism, potential differences in how depression may present in autistic vs. non-autistic populations, and conceptual considerations for research on co-occurring autism and mental health challenges. We then provide a summary of cognitive, developmental, and clinical factors that have been identified through previous studies as important contributors to elevated rates and severity of depression in autistic individuals. Several, often closely interrelated, factors may affect the development and maintenance of depression in this population, including core autism features as well as cognitive and emotional experiences that are common in autistic individuals but not part of the diagnostic criteria for autism. Further, we propose a conceptual integration of the noted factors, specifying potential patterns of their interrelatedness. Finally, we put forward conceptual and empirical strategies for formally testing and refining our proposed model, and describe its implications for research, clinical practice, and societal reform.

## 1. Introduction

Co-occurring mental health challenges are critical to consider when evaluating and implementing clinical support to improve concurrent and long-term quality of life and functioning outcomes in autistic individuals (although language preferences surrounding autism vary from person to person, the authors have elected to use identity-first language for this review, as a growing body of autistic adults have indicated that they prefer it over person-first language). Autistic people are disproportionately likely to experience mental health challenges during their lifetime, with anxiety and depressive disorders being particularly common. A multi-country meta-analysis indicated that current and lifetime rates for depressive disorders in autistic individuals are approximately 23% and 37%, respectively [1]. Moreover, estimates of diagnosis prevalence have indicated that approximately 11% of autistic individuals had a clinical diagnosis of some form of depressive disorder [2]. This is in stark contrast to the World Health Organization’s general population prevalence rate for depression in 2019, estimated at 3.8% for the global population [3]. The disparity in these prevalence estimates suggests that depression is a particular concern in the autistic population.

Mental health challenges, such as depression, may manifest differently in autistic individuals than in their allistic (non-autistic) [4] counterparts. For example, an estimated 50% of autistic people have co-occurring alexithymia [5], which may make self-reporting mood more difficult. Others, particularly those who are non-verbal and/or have co-occurring intellectual disabilities, may be unable to self-report on mental health. Therefore, diagnosis of depression in autistic individuals often requires actively engaged informants to report on affective symptoms [6]. In addition, signs of depression (e.g., loss of interest in social interactions, self-injurious behavior) may coincide with core autism diagnostic criteria, and increases or decreases in specific restricted and repetitive behaviors (e.g., reduction in special interests) may often not be correctly attributed to depression [6]. Finally, the majority of instruments designed to diagnose depression or quantify symptomology have been developed for allistic populations and may mis-estimate atypical expressions of depression symptoms in autistic individuals [7,8]. For instance, loss of self-care skills is relatively common in autistic individuals experiencing depression but may not be explicitly screened for when assessing depressive symptomatology [6]. A combination of such factors often results in autistic individuals’ depressive symptoms being missed due to diagnostic overshadowing, in which depressive symptomatology is incorrectly attributed to the primary autism diagnosis [1].

Untreated depressive symptoms in autistic individuals can have severe consequences, with one of the most concerning being the elevated rates of suicidal thoughts and attempts among autistic adults. One study conducted at a specialist diagnostic clinic found that 35% of autistic participants had either planned methods of suicide or made an active suicide attempt, and 66% of their sample had experienced suicidal ideation [9]. Therefore, research directed towards the identification of factors contributing to the onset and progression of depressive symptoms in autistic individuals could help shape clinical approaches to mental health treatment and suicide prevention.

While the body of literature concerning depression in autistic individuals is steadily growing, much of the research remains descriptive, focused on group-level comparisons between autistic and allistic individuals with respect to depression prevalence and predictors. Further research is needed to understand more fully the complex array of factors that contribute to elevated rates of depression among autistic individuals, exploring how these factors may be modified by individual difference and environmental factors to produce heterogeneous outcomes. The objective of this narrative review is to comprehensively survey the available literature to identify processes associated with depression in autism that may guide future systematic review efforts and to propose a hypothesized model for subsequent empirical testing. We provide an overview of existing literature, highlight current gaps, and conclude with a discussion of future directions for research and clinical practice in this area. Given the narrative design, the review does not aim for exhaustive coverage but instead provides a structured, conceptually focused synthesis intended to guide future systematic and empirical work. A table indicating all studies reviewed concerning autism–depression associations is available in the Supplementary Materials.

## 2. An Integrated Model of Factors Contributing to Depression

**Figure 1:**
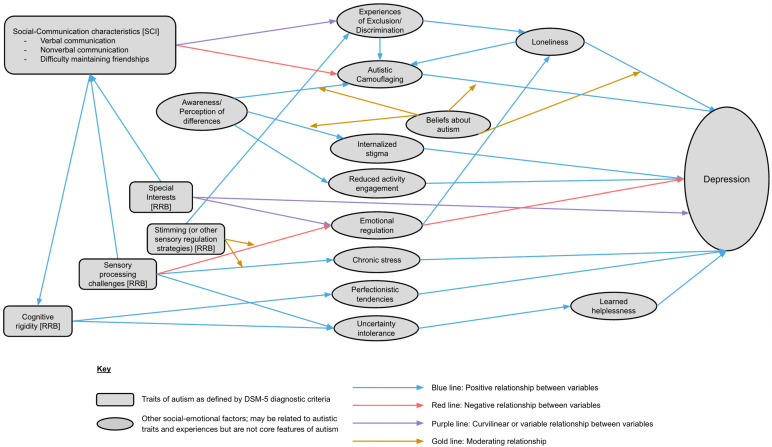
An Integrated Model of Direct and Indirect Factors that may Influence Development of Depression in Autism.

Figure 1 and Table 1 illustrate our proposed model of factors contributing to the relationship between autism and depression. These include autistic traits as defined by the Diagnostic and Statistical Manual for Mental Disorders as well as social-emotional factors related to autism but not a part of the core diagnostic criteria. Exclusion and discrimination may present direct pathways to depression or may be mediated by loneliness [10,11]. Loneliness, exclusion, and discrimination may also lead to increased tendencies to camouflage autistic traits, which may further contribute to risk of depression [12,13,14]. Awareness and perception of personal differences may also contribute to depressive symptomatology by increasing a sense of internalized stigma around autism, and by decreasing participation in meaningful activities [15]. Given previously inconsistent literature, with some studies indicating positive associations between communication difficulties and peer victimization, but others reporting negative or null relations between autism-related social impairment and victimization [16,17,18], we hypothesize that the relationship between autistic traits and exclusion may be curvilinear, such that those with moderate degrees of social impairment will face the most exclusion and discrimination.

Restricted and repetitive behavior (RRB) features, such as cognitive rigidity, sensory processing challenges, special interests, and stimming, each have distinct relationships with depression in autistic individuals. Cognitive rigidity is associated with increased intolerance of uncertainty, executive functioning challenges, and maladaptive perfectionism, all of which could increase risk for depression [19,20,21,22]. Sensory processing challenges may lead to chronic overwhelm and social withdrawal [23,24]. Stimming has potential to moderate the relationship between sensory overwhelm and chronic stress but may also lead to exclusion and discrimination in unsupportive environments [25,26,27]. Special interests could lead to a sense of fulfillment and joy from engagement but may also become obsessive and detract from the ability to complete self-care activities [28,29,30]; therefore, the relationship between special interests and mental health is hypothesized to be curvilinear.

Several key moderators may influence the pathways described above. Beliefs about autism are hypothesized to moderate relationships between many of the socially influenced processes [31,32,33]. Neutral or partially positive (rather than wholly negative) beliefs about autism may serve as a protective factor against internalized stigma, allow individuals to adopt more pragmatic and less self-disparaging views about why they choose to camouflage their autistic traits, and help lonely autistic individuals to believe that their loneliness is temporary and modifiable, rather than an immutable aspect of their life [33]. Finally, emotion regulation capability affects presentation of autistic traits, and also influences others’ perceptions of an autistic individual.

As highlighted above, there is an emerging evidence base suggesting that depression is associated with both core traits of autism and with additional social-emotional factors that are not core traits of autism. Previous studies on autism have focused predominantly on understanding either one or the other of these associations; however, both have substantial impact, and interactions between factors may differentially influence the risk of depression. The present model aims to advance understanding of co-occurring autism and depression by comprehensively synthesizing multiple key factors across these categories and proposing potential interrelations between autistic traits and other social-emotional experiences to be explored in future empirical research.

The aforementioned model should be considered an integrative, preliminary model, rather than an exhaustive list of possible variables at play. Relationships between autism and depressive symptomatology are evidently complex, and future research may identify additional salient factors influencing this relationship. However, the model provides a summary of previously identified factors, in addition to proposing new avenues for exploration. Subsequent sections of the article review theoretical considerations in autism research, review literature supporting the current integrative model, and highlight conceptual relations within the hypothesized pathways.

**Table 1:** A Summary Table of Factors Associated with the Development of Depression in Autistic Individuals.

Factor	Correlation with Depression (Positive, Negative, Curvilinear)	Key References
Core autism traits as defined by the DSM-5 diagnostic criteria		
Social-Communication characteristics	Curvilinear	[34,35,36,37,38,39]
Special interests	Curvilinear	[28,29,30]
Stimming	Curvilinear	[26,27]
Sensory processing challenges	Positive	[23,24,40,41,42]
Cognitive rigidity	Positive	[21,43]
Other social-emotional factors		
Experiences of exclusion/discrimination	Positive	[13,16,17,18]
Autistic camouflaging	Positive	[44,45,46,47]
Loneliness	Positive	[12,13,14]
Beliefs about autism	Moderator; negative thoughts about autism are positively associated with depression while positive thoughts about autism may protect against depression	[31,32,33]
Internalized stigma	Positive	[10,11]
Reduced activity engagement	Positive	[15]
Chronic stress	Positive	[48]
Perfectionism	Positive	[20]
Uncertainty intolerance	Positive	[41,42]
Learned helplessness	Positive	[19]

## 3. Methods

This narrative review was developed in accordance with principles outlined in the Scale for the Assessment of Narrative Review Articles (SANRA) quality appraisal tool [49] to enhance clarity, rigor, and balanced presentation. Initial literature searches were conducted in APA PsycINFO and Google Scholar in October 2023 to identify factors relevant to depression in autism. After the initial set of factors was identified, additional targeted PsycINFO searches were conducted using terms related to these processes. Searches revealed publications from 2003 to 2025 using combinations of the following terms: “autism”, “autism spectrum disorder”, “depression”, “autistic special interests”, “stigma”, and “autistic camouflaging”. Relevant work cited in papers from this initial review were accessed, as were newer articles citing these papers. A narrative synthesis approach was used to iteratively organize and integrate themes across the literature. Given the narrative nature of this review, inclusion was guided by conceptual relevance rather than strict methodological criteria. Only empirical articles were considered, with no restrictions on research design. Articles were included if they contributed meaningfully to understanding processes associated with depression in autistic individuals. Literature from adjacent domains (e.g., depression processes studied outside autism) was included when it informed conceptual understanding. Exclusion was limited to sources that did not address autism, depression, or the processes under consideration.

## 4. Positioning the Integrated Model in Relation to Medical and Social Models of Disability

Research on autism and co-occurring mental health challenges, including depression, can be engaged with from multiple theoretical perspectives. The following section provides a brief definition of both the medical and social models of disability and provides context for how the current integrated model draws upon both perspectives. In the research and medical communities, autism is most commonly characterized as a neurodevelopmental disorder that profoundly affects social communication and behavior [40]. Social-communication and interaction (SCI) difficulties and the presence of restricted and repetitive patterns of behaviors and interests (RRB), while both core aspects of autism, may make unique and additive contributions to mental health outcomes in autistic individuals. Although previous research demonstrates the clear separability of the SCI and RRB domains, and suggests that they have different genetic, neural, and cognitive origins [50], there are interrelations among SCI and RRB in prediction of outcomes [51]. Additionally, each broad domain encompasses a range of distinct subdomains with at least partially distinct trajectories and potential mechanisms for affecting mental health outcomes. Therefore, considering specific SCI and RRB subdomains is essential for gaining more nuanced and accurate insights into mechanisms underpinning depressive symptomatology.

An alternative perspective on autism is derived from the social model of disability. Autistic individuals can be thought of as members of a minority social group with its own loosely defined culture (including values, customs, and group identity) [52]. For instance, some scholars have drawn parallels between autistic culture and Deaf culture [53,54]. As such, the social world, perceptions of autism, and discrimination have salient effects on autistic individuals’ wellbeing, as they do on members of other minority groups (including racial and cultural minorities as well as physically disabled individuals) [55,56,57]. The Minority Stress Model is highly applicable when studying mental health outcomes in autistic individuals, as they may encounter overt discrimination, internalized stigma, concealment, and other forms of minority stress throughout their lifetime [10,34]. A study applying the Minority Stress Model in autistic populations found that a variety of stressors predicted psychological distress in autistic individuals, including everyday discrimination, and expectation of rejection, outness, and internalized stigma [10]. Most notably, they found that a formal autism diagnosis predicted lower psychological wellbeing and greater psychological distress. They suggest that this might be due to the opinions and stereotypes that others impose on an autism diagnosis, rather than due to individuals’ own opinions of their diagnosis. A follow-up study found that, while autistic people were most likely to see their autism as value-neutral, they struggled with the negative valence that people around them often applied to their autism diagnosis [10].

In summary, both autistic traits and broader societal perception of autistic traits are important to consider when researching mental health in this population, and the following review aims to account for both medical and social perspectives. In addition to exploring the direct effects of autistic traits as defined by the Diagnostic and Statistical Manual for Mental Disorders (i.e., SCI and RRB), it additionally integrates societal perceptions and structural barriers as factors that can further amplify already-existing challenges. The following sections elaborate on processes that link SCI and RRB traits to depression, alongside relevant broader societal factors.

## 5. Associations Between Social-Communication and Depression

There is a pronounced heterogeneity among autistic individuals with regard to specific social communication and interaction (SCI) traits. Indeed, several studies have identified subgroups of autistic individuals with profiles of strengths and weaknesses across distinct social and communication subdomains, including differing levels of social motivation and ability to recognize and interpret social signals, and social skills [35,36,37,38,58]. Therefore, it follows that each individual experiences different risk and protective factors for depression in relation to their SCI traits. However, clear associations emerge between certain clusters of difficulties and depression, including challenges with making friends and self-perception of SCI traits. Additionally, interactions with the surrounding environment may further amplify the effects of these challenges.

When considering SCI traits, the often-substantial social-communication differences between autistic and allistic individuals may contribute to a mutual lack of understanding between parties, contributing to difficulty initiating or maintaining friendships over time [39]. Lack of high-quality friendships has been associated with increased rates of depression in autistic people [59,60]. This may particularly be the case if an autistic individual’s intense interest(s) are not reciprocated by others around them, as friendships of both autistic children and adults are often built heavily around mutual interests [29,60]. Social motivation, social skills, and social cognition could interact with each other to influence outcomes of friendship and exclusion. For example, increased or typical levels of desire to form social relationships, combined with differences in social skills and social cognition, could lead to a lack of reciprocal friendships. Repeated challenges forming friendships could then lead to isolation and loneliness.

Lack of success following atypical social approaches could also alter self-perception. In a sample of clinically diagnosed autistic children and adolescents, perceived group membership correlated negatively with depressive symptomatology. In the sample studied, awareness of personal differences and negative self-perception of autistic differences were associated with depressive symptoms in autistic individuals [15]. Additionally, intrinsic differences in social interaction may affect emotional regulation—for individuals who have ideas or needs they are trying to convey to others but are being misunderstood, the disconnect between their communication and the others’ understanding may lead to frustration, overwhelm, or shutdown [61]. Repeated instances of communication-related frustration may in turn affect self-perception and self-efficacy [15].

Although the mechanisms described above hinge on the presence of autistic traits, some of these mechanisms also implicate broader social processes, such as overt and subtle discrimination and victimization from peers. These minority stressors [34] do not occur merely because individuals are autistic but rather because of third-party responses to autistic traits. Therefore, many social-communication risk factors for depression in autistic individuals could be substantially mediated by the social environment around them. The following subsections further examine aspects of the social environment in relation to depression.

### 5.1. Experiences of Discrimination, Exclusion, and Alienation

In addition to encountering more challenges when forming positive peer relationships, autistic individuals may also face increased exclusion and alienation from peers, which may confer greater risk of developing mental health challenges like depression. School-age autistic children are significantly more likely to experience bullying or victimization at school than their allistic counterparts [62]. Social communication has been frequently explored as an individual factor that contributes to school victimization, with research producing conflicting findings. Whereas some studies found that greater social-communication difficulties were associated with greater victimization, some found no meaningful relationship between the two variables, and others found the opposite pattern [16,17,18].

While it is possible that these studies yielded different results due to differing measurement of constructs, as the authors of one of the aforementioned studies suggested [62], the nature of the samples may have also played a role. For example, in studies that found that children with fewer social-communication difficulties were more likely to be victimized, allistic children may have been taught that it is inappropriate to make fun of autistic peers with severe associated disabilities but may feel fewer qualms about victimizing a peer with fewer overt differences. A curvilinear pattern could thus explain discrepancies in previous results. Those with a moderate degree of social impairment may experience the most victimization, as they may be able to participate in peer social interactions but not conceal their social challenges. Those with mild and severe impairments may experience comparatively less victimization but for different reasons. Those with mild impairments may be able to blend in with their peers, while those with the most severe impairments may be more carefully protected by their teachers and peers. While this hypothesis has yet to be formally tested, understanding circumstances in which autistic students with varying levels of support needs may be at greater risk of bullying could help schools intervene more effectively when bullying is suspected.

Exclusion and alienation of autistic individuals continue into adulthood, albeit usually in less overt ways. In one experimental study, allistic study participants were asked to evaluate the personality characteristics of target autistic and allistic individuals. Despite the fact that participants were not aware of individuals’ diagnostic status, neurotypical participants rated autistic individuals as being less approachable, less friendly, and more awkward [13]. The results of this study indicate that allistic individuals are often instantaneously and subconsciously able to differentiate autistic individuals from their allistic counterparts and may choose to interact less with the autistic individuals due to these judgements. Such social exclusion processes may further contribute to the loneliness that autistic individuals experience throughout the course of a lifetime. In autistic adults, measures of autistic traits have been found to significantly positively correlate with loneliness and indirectly predict depression [12,14].

Taken in sum, these studies suggest that social processes such as loneliness, exclusion, and discrimination play a substantial role in mediating the relationship between autistic social-communication traits and depression. With these aversive social processes at play, some individuals may diminish or hide their expression of autistic traits to attempt to avoid social scrutiny and thereby protect their mental health; however, such strategies may have their own additive effects on the relationship between autism and depression.

### 5.2. Autistic Camouflaging

Autistic camouflaging, alternatively referred to as compensation, masking, and/or adaptive morphing, has been defined as “the employment of specific [behavioral] and cognitive strategies by autistic people to adapt to or cope within the [predominantly] non-autistic social world” [44]. Several of the factors named above—difficulty with building and maintaining friendships, overt victimization or exclusion from peers, self-perception of autistic differences, and loneliness—may all contribute to autistic individuals choosing to camouflage their traits. While individuals who camouflage may find it beneficial for social or work situations, camouflaging takes a substantial amount of vigilance and cognitive effort, and frequently leads to exhaustion, stress, and a feeling of not being authentically known [45].

Studies leveraging self-reported, clinician-reported, and caregiver-reported camouflaging converge in suggesting that autistic camouflaging may contribute to adverse mental health outcomes. In one study of autistic adults, self-reported camouflaging of autistic traits was found to be significantly related to depression, generalized anxiety, and social anxiety for both men and women [46]. Another study measured camouflaging through obtaining the difference between clinician-observed and self-reported autistic traits rather than exclusively through self-report, and found a significant effect of camouflaging on depression, but only in men [47]. In a comparison of adolescent self-reports and parental reports regarding camouflaging, researchers found that adolescents who display more prominent self- and caregiver-reported indicators of autism and generalized anxiety engage in more camouflaging behavior, which may in turn contribute to worse mental health [63].

In addition to the direct pathway from social isolation and loneliness to depression, camouflaging may create an indirect pathway for autistic individuals. When confronted with the experience of social isolation or loneliness, an autistic individual may choose to camouflage their traits in the hopes of finding a sense of belonging in a group. However, as outlined above, the negative effects of camouflaging may lead to greater depressive symptoms [2,46]. Thus, autistic camouflaging might serve as a mediator of the relationship between loneliness and depressive symptoms.

In summary, although autistic camouflaging is intended to be adaptive for increasing emotional wellbeing through bolstering friendships and workplace communications, it may have unintended adverse outcomes for mental health.

### 5.3. Beliefs About Autism and Autistic Social Identity

One factor that potentially moderates the relationship between autism, loneliness, and depression is the degree to which people positively identify with a sense of autistic identity. While research in this area is still emerging, several studies suggest that a positive sense of autistic identity can protect against depressive symptoms. One indicated that autistic individuals had significantly poorer mental health than their allistic peers, autistic individuals who reported a strong sense of ‘autism identification’ showed fewer depression symptoms than those who did not [33]. Another study found similar results in an autistic-only sample: socially identifying with other autistic individuals, but not with family or other activity groups, significantly predicted fewer depressive symptoms [32]. The opposite also appears to be true: that feeling exclusion or dissatisfaction related to an autism diagnosis predicted significantly lower self-esteem and wellbeing [31]. Based on the current body of research, individuals’ sense of autistic identity is a valuable avenue for future study when considering the relationship between autism and depression.

Considering these studies alongside those applying the Minority Stress Model to autism, it seems that there may be benefits to developing a positive self-identity in relation to autism; however, social stigma, externally imposed stereotypes of autism, and other minority stressors may make the formation of a positive autism-related self-identity difficult. The complexity of beliefs about autism, both internal and external, is crucial to consider in future research and clinical work focusing on depression in autistic individuals.

## 6. Associations Between Restricted and Repetitive Behaviors and Depression

Restricted and Repetitive Behavior (RRB) traits refer to a cluster of non-social characteristics of autism, including sensory sensitivity, behavioral and cognitive inflexibility, self-stimulatory behavior (stimming), and special interests. Each of these traits has its own unique implications for depression in autistic individuals. Behavioral and cognitive inflexibility, defined as compromised ability to switch cognitive models to adjust behavior to changing demands [43], are commonly observed in autism, and importantly, have been found to be associated with greater risk for depression among autistic individuals, as well as for the general population [21,48]. Research suggests that cognitive inflexibility increases likelihood of rumination and decreases the ability to solve problems in a flexible manner, which may create a potential pathway to depression. In autistic individuals, cognitive inflexibility has been found to be positively associated with both internalizing and externalizing symptoms [21]. Degree of cognitive flexibility has also been found to serve as a moderator between stressful family events and depression in autistic individuals, such that having greater cognitive flexibility minimized the impact of aversive family events on depression [64].

Additional factors may further mediate the relationship between cognitive inflexibility and depression for autistic individuals. Cognitive inflexibility is closely related to executive function, which can make it more difficult for autistic individuals to complete other essential daily life activities, conferring additional risk for depression [41]. Additionally, several studies have found that cognitive inflexibility is related to lower uncertainty tolerance [21,22]. While uncertainty tolerance is often applied to anxiety rather than depression, the feeling that the world is deeply unpredictable may lead to a sense of learned helplessness in autistic individuals [19], which could mediate the relationship between cognitive inflexibility and depression. Further, clinical perfectionism (e.g., holding rigid and overly punitive ideas about the ‘correct’ and ‘incorrect’ ways to do things) was found to mediate the relationship between cognitive inflexibility and both depressive and anxiety symptoms in a sample of autistic boys [20].

Sensory processing challenges may additionally contribute to difficulty completing daily life activities in autistic individuals. In younger autistic children, hypersensitivity to sensory stimuli has been found to significantly correlate with both internalizing and externalizing problems [24]. Additionally, sensory sensitivities are associated with chronic stress for autistic individuals, and that socially sensitive individuals may potentially cope with sensory overload by withdrawing from social settings and enjoyable activities [23]. Both the chronic stress associated with sensory overwhelm and the resultant withdrawal could mediate the relationship between sensory processing challenges and depression. Additionally, research has linked both sensory hypersensitivity and hyposensitivity to uncertainty intolerance, with some studies suggesting that sensory processing challenges indirectly predict greater uncertainty tolerance [34,42,65].

The relationship between autistic self-stimulatory behavior (stimming) and depression is less straightforward than the presumed impact of cognitive inflexibility and sensory processing challenges. One study found that both autistic and allistic adults reported positive experiences with stimming, describing it as a way to self-regulate and successfully handle otherwise overwhelming sensory input [26]. For autistic individuals, stimming can be relaxing or enjoyable in its own right: up to 80% of autistic adults reported that they generally enjoyed stimming, with another 11% reporting that it depended on the particular stim [27]. However, the environmental response to stimming may differ widely based on the peer group of an autistic individual. For some autistic individuals, stimming may lead to corrective feedback, harsh criticism, or open exclusion, particularly if their stims are perceived as “distracting” or “weird” to the people around them [25,26], potentially contributing to social exclusion of autistic persons. Thus, stimming may moderate the relationship between sensory sensitivity and emotion regulation, and the relationship between sensory sensitivity and chronic stress, while concurrently conferring greater risk of discrimination and exclusion in an unsupportive environment.

Autistic special interests hold a complex relationship to mental wellbeing. Autistic special interests could be hypothesized to reduce an autistic individual’s likelihood of depression by providing a sense of joy and personal fulfillment, as self-reported by many autistic individuals [30]. Special interests may, in addition, be a viable and comfortable avenue through which autistic individuals can build meaningful and lasting friendships with other individuals, both autistic and allistic, who have overlapping interests [28,29]. However, special interests could also become perseverative or obsessive, which may detract from social interaction or the ability to complete other activities necessary for maintaining good physical and mental health. As such, a curvilinear mediating relationship may exist between special interest engagement and depressive symptoms, such that there is an optimal quantity and quality of special interest engagement for promoting positive mental health outcomes in autistic individuals. Measures already exist to quantify the quality and purpose of special interest engagement [66] but may not provide sufficiently nuanced understanding of adaptive and maladaptive special interest behavior. Additional qualitative research to understand how autistic individuals experience their special interests may be beneficial in creating more comprehensive models of special interest engagement as related to mental health.

In summary, some RRB traits appear to be linked to an increased risk for depression, while others appear to be neutral or even positive in relation to an autistic person’s overall mental health. Cognitive rigidity and sensory processing challenges have been found to be frequently associated with depression, while special interest and self-stimulatory behavior may serve as a protective factor or a risk factor for depression depending on an individual’s social environment and individual characteristics. Whenever possible, measuring these traits separately rather than in aggregate will likely yield the most accurate understanding of the role that each play in mental health outcomes for autistic individuals.

## 7. Links Between Social-Communication and Restricted and Repetitive Behaviors

In addition to relationships established above, specific facets of Social Communication and Interaction (SCI) and Restricted and Repetitive Behavior (RRB) domains may be strongly interrelated with each other. For instance, previous research has suggested that social motivation in autistic individuals tends to be diminished due to greater attentional resources being directed toward non-social areas of interest [67]. Sensory features may also influence atypical social approaches [68].

Interrelations between SCI and RRB traits may make it more difficult to determine the exact contributions of each trait to depression. In studies of autism and depression, it is important to consider the potential influence of other SCI and RRB traits outside of those in the immediate model being tested, particularly when evaluating strengths and limitations of existing measures and when developing new studies. Additionally, these complex interrelations suggest that certain combinations of SCI and RRB traits may interact with each other and confer greater risk of depression. Existing research on subtypes of social-communication and RRB in autism has already suggested certain profiles, such as the ‘active-but-odd’ profile [58], may be more prone to internalizing symptoms [69]. Further research could explore the exact mechanisms by which these traits interact to influence mental health. 

## 8. Direct and Indirect Associations Between Emotion Regulation and Depression

Emotion regulation is a major transdiagnostic factor that may influence both presentation of autistic traits and likelihood of developing depression. Weaker skills in emotion regulation are associated with reduced social skills and fewer instances of prosocial behavior in autistic children [70,71]. In both general population studies and autism-specific studies, lower ability to regulate emotion has been found to negatively affect abilities to form and maintain rewarding friendships [72,73]. Additionally, autistic children who overtly express dysregulation were found to be more likely to stand out to their peers as vulnerable, ending up at greater risk of both victimization and loneliness [74,75,76]. Intervention work in this area indicates that building skills to manage challenging emotions can result in greater social interaction ability, suggesting a directional relationship in which emotion regulation acts on other processes [77].

In addition to its effects on social processes, emotion regulation also affects the relationships between RRB traits and depression. It has been found to mediate the relationship between sensory processing challenges and both internalizing and externalizing problems [78]. For those who are less able to cope with their emotions, they may experience increased distress in response to situations that cause sensory overwhelm. Difficulty with managing emotions is also associated with greater rumination, which is in turn associated with greater depressive symptomatology [79]. As highlighted previously, special interests and stimming may provide outlets through which individuals can successfully regulate and ground themselves. The existing body of literature indicates the overarching influence that emotion regulation has on mental health outcomes in autistic individuals and suggests that it may be a continued area of focus for future studies. In summary, emotion regulation may have important implications for depression in the autistic population, including for intrapersonal and interpersonal development, and could be an important area for further study.

## 9. Future Directions

The proposed integrative model has relevant applications across multiple domains of research and applied practice focusing on autistic individuals. The final sections of the paper explore potential implications for various aspects of the model in regard to research, clinical practice, and societal support and acceptance for autistic individuals who are currently experiencing or are at risk of experiencing depression.

### 9.1. Research Implications

The model presented herein is a starting point to guide further tests of relationships between the processes linking autistic traits and depression. An ancillary goal is to encourage consideration of additional pathways and moderators. One area for future research is exploration of relations between an autistic individual’s degree of social difference and the degree to which they experience exclusion and discrimination. Given the discrepancies in previous results, a curvilinear relationship may help to explain contradictory findings in prior studies. There may additionally be factors that play a moderating role in this relationship, such as other parties’ understandings of autism and local culture surrounding social relationships. Another area that requires further study is the relationship between autistic special interests and depressive symptomatology. Because special interests can be a central part of an autistic person’s experience, understanding how special interests are experienced could provide crucial insight into mental health in autistic people [30].

A variety of research methods could be used to enhance understanding regarding co-occurring autism and depression. A limitation of many clinical studies is reliance upon retrospective and global self-report of symptoms. Future studies could employ methods such as ecological momentary assessment to obtain more accurate and nuanced data on the impact of autistic traits, and of external influences, on mental health [80]. Additionally, many existing studies focusing on mental health in autistic individuals collected cross-sectional data, limiting clarity on causal direction of relationships between relevant contributing factors. Future studies using longitudinal designs could provide additional insight on directionality of relationships. Finally, while the body of research on autism-related behaviors and cognitions is steadily increasing, there are still many aspects of autistic experiences that remain underexplored. Further qualitative research centering autistic individuals is necessary in order to develop autism-specific measures relating to depression, other mental health challenges, and healthy and unhealthy coping mechanisms, with a particular emphasis on emotional regulation.

Research on depression interventions for autistic individuals would also be enhanced when situated in the context of a theoretical model. The present model proposes that some autistic behaviors, such as stimming and engaging with special interests, could provide pathways to more optimal mental health in autistic individuals, provided that they are in a supportive environment where such behaviors are accepted non-judgmentally. If evidence supports this proposal, clinical researchers could develop and refine mental health interventions focusing on promotion of autism-specific adaptive behaviors. These interventions could be used independently, or in conjunction with existing depression treatments, based on individuals’ needs.

### 9.2. Clinical Implications

Integrative models such as this one also have high relevance for clinicians treating depression in autistic individuals. While aims to reduce clients’ expression of autistic traits in the service of diminishing depressive symptoms may initially appear to be an acceptable therapeutic goal, the current literature does not support the success of this approach. In contrast, techniques targeting autistic behaviors, such as Applied Behavioral Analysis, have been demonstrated to encourage camouflaging of autistic traits and exacerbate mental health symptoms [81,82]. Adverse effects of such therapies may relate heavily to experience of minority stressors, particularly internalized stigma. Constant correction for displays of autistic behaviors may contribute to feelings that being autistic is unacceptable. Any therapy that does not take into account social factors outside the autistic individual—such as discrimination and exclusion from others, and others’ beliefs about autism—as potential contributors to depression should be considered an incomplete approach, and potentially detrimental to mental health outcomes.

Emerging literature suggests that evidence-based therapies such as Cognitive Behavioral Therapy and Dialectical Behavior Therapy may be effective for treating depression in autistic clients, particularly when adapted for their sensory and social needs [83,84]. In addition, strengths-based and neurodiversity-affirming approaches are increasingly gaining traction [85]. These approaches could provide opportunities to encourage self-acceptance, reconnect with individual strengths, and reframe existing negative beliefs about autism. Clinicians could also use neurodiversity-affirming therapy spaces to help autistic clients set sustainable and personally meaningful goals, such as reducing day-to-day camouflaging behavior, connecting with others in a sincere way, and learning new strategies for sensory regulation. Clinicians who work with autistic clients (regardless of whether they themselves are allistic or autistic) must take special care to select treatment approaches that are appropriately adapted for their clients’ needs, avoid inadvertently endorsing stigma about autistic traits, and stay up to date on literature regarding best practice in mental health care for autistic individuals.

### 9.3. Societal Implications

Beyond providing a framework for future research and suggestions for clinical applications with autistic clients, the model proposed herein suggests value in developing non-stigmatizing and neurodiversity-affirming psychoeducation about autism for caregivers, peers, and colleagues of autistic individuals. It is important to note that system-level barriers (such as stigma and lack of service access) are modifiable risk factors, and that taking steps to address them may have substantial benefits to mental health in this population. Given the large role that the environment may play in autistic individuals’ mental health, such interventions targeted at the broader social sphere would be a useful supplement to treatment of individual autistic persons.

A promising avenue for reduction and management of depressive symptomology among those with autism involves efforts to enhance familial support. Previous research indicates that parent–child interactions can help to facilitate the development of social skills over time [86], which may help autistic children to build a greater number of lasting friendships. Autistic individuals can also find a sense of belonging within a supportive family, particularly if they have other autistic family members [87], which reduces feelings of loneliness even when friendships are not particularly robust. Finally, although not previously tested empirically, families of autistic children could influence a child’s development of a positive autistic self-identity, which could serve as a further protective factor against depression. 

Further, support in a school context also has important effects on academic performance and sense of belonging for school-aged autistic children and adolescents. Comprehensive support from an interdisciplinary team at school (including school psychologists, teachers, and occupational therapists) can help autistic students to meet major academic and social milestones in a timely manner [88], which may reduce their likelihood of being excluded or victimized by their peers. School-based social support and interventions can also help autistic children to receive accommodations that reduce the chance of sensory or cognitive overload [89]. These interventions could prevent chronic exhaustion and stress from unmet sensory needs and may reduce the chance that depressive symptoms will develop. Finally, cognitive–behavioral counseling is becoming an increasingly common part of Individualized Education Plans for autistic students, with one study indicating that up to 44% of autistic students have some form of counseling as part of their Individualized Education Plan [90]. Mental health outcomes research examining the effects of multidisciplinary support, academic or social accommodations, and school-based counseling could be used to further develop the evidence base for the use of comprehensive support at school for autistic children and adolescents.

Finally, although research on support resources for autistic adults—particularly older autistic adults—is limited, the existing literature suggests the continued importance of support networks for autistic adults. A preliminary study found that both objective measures of social support and subjective feelings of being supported increased older autistic adults’ overall quality of life and decreased both depressive and anxiety symptomatology [91]. Future research could explore additional novel methods of building and maintaining support networks for autistic adults, including through in-person and online platforms. By leveraging research and clinical practice to shift broader societal attitudes toward autism, and to increase acceptance and support for autistic individuals, further-reaching changes can be made to empower autistic individuals in maintaining wellbeing and avoiding depression at all stages of their lives.

## 10. Limitations

Through a broad survey of the existing literature, this review highlights a number of pertinent processes associated with depression. Nonetheless, this review is narrative in nature and does not employ a systematic or exhaustive search strategy. Literature was selected based on conceptual relevance, which may introduce selection bias, and searches were limited to specific databases (APA PsycINFO and Google Scholar), potentially excluding relevant studies elsewhere. Only empirical articles were included, without restrictions on research design, resulting in variability in study quality and methodology. The narrative synthesis approach relies on interpretive integration of heterogeneous studies and does not allow for quantitative comparisons or effect size estimates. Additionally, the review focuses on select processes associated with depression in autistic individuals and may not encompass all contributing factors. Consequently, the findings should be interpreted as conceptual guidance for future research rather than definitive conclusions about causal relationships.

## 11. Conclusions

Through a broad survey of the existing literature, this narrative review highlights processes associated with depression in autism that warrant focused systematic review efforts and advances a hypothesized model for evaluation in future empirical studies. The conceptual model proposed in this review integrates core autistic traits—including Social Communication and Interaction (SCI) differences and Restricted and Repetitive Behaviors (RRB), and their interrelations—in addition to other social-emotional factors related to autism.

Our hypothesized model proposes that direct connections between autistic traits and depressive symptomatology are likely complemented by mediating and moderating processes including experiences of exclusion or discrimination, loneliness, autistic camouflaging, and perfectionism. Pertinent areas of future study include the confirmation of relationships between autistic traits, depression, these factors, and on the development of tailored psychosocial and broader community-focused interventions to prevent and treat depressive symptoms in autistic individuals.

## Data Availability

No new data were created or analyzed in this study.

## Supplementary Materials

The following supporting information can be downloaded at: https://www.mdpi.com/article/10.3390/healthcare13233112/s1, Supplementary File: Supplementary Table S1 of Studies Reviewed Regarding Autism–Depression Associations [10,14,15,20,21,23,24,26,27,28,30,31,32,33,41,44,46,47,51,59,63,64,70,71,73,74,76,78,79,87,91,92,93,94].
