# Supplementary material for: Processes Associated with the Development of Depression in Autistic Individuals: A Narrative Review
Source: Healthcare (Basel). 2025 Nov 28;13(23):3112. doi: 10.3390/healthcare13233112 (PMC12692341; doi:10.3390/healthcare13233112)
Supplement: Supplementary file 1 [file healthcare-13-03112-s001.zip › healthcare-3977054-supplementary.pdf]

Supplementary Table S1 of Studies Reviewed Regarding Autism-Depression Associations

**First author**	**Design**	**Participant/ Reviewed Studies Characteristics**	**Key Measures**	**Explanatory variable(s) explored**
Bitsika [23]	Quantitative, cross-sectional	Autistic girls and adolescents (n = 53)	*Autistic traits:* Prior formal diagnosis of autism (reported by parent)<br>*Depression:* Child and Adolescent Symptom Inventory-MDD subscale (parent-report and self-report)<br>*Sensory sensitivity:* Sensory Profile (SPr) (parent-report and self-report)	Sensory sensitivity
Black [28]	Scoping review of qualitative studies	Qualitative or mixed-methods studies (n = 22), focusing on autistic friendships with other autistic individuals and/or with neurotypical individuals	n/a— systematic review	Friendship/ inclusion
Bohnert [76]	Quantitative, cross-sectional	Dyads of autistic children and their parents (n = 127)	*Depression:* Achenbach Child Behavior Checklist (parent-report), Achenbach Youth Self Report (self-report)<br>*Loneliness:* Loneliness Scale<br>*Friendship quality:* Friendship Quality Questionnaire - Abbreviated Parent edition and Friendship Quality Questionnaire - Abbreviated edition.<br>*Organized Activity Involvement:* Organized Activities Inventory	Loneliness, Organized Activities Involvement
Botha [10]	Quantitative, cross-sectional	Autistic adults (n = 142)	*Discrimination:* Everyday Discrimination Scale<br>*Rejection:* Experiences of Rejection Scale<br>*Internalized Stigma:* Adapted from Meyers and Dean 1998 for autism<br>*Psychological Distress:* Kessler 6-item Psychological Distress Scale	Exclusion, discrimination, internalized stigma
Cappadocia [74]	Quantitative, cross-sectional	Parents of autistic children (n = 192)	*Child autistic traits:* Autism Spectrum Quotient-50 (parent-report)<br>*Bullying experiences:* Promoting Relationships and Eliminating Violence Network Assessment Tool—Parent Version	Social-communication characteristics, social exclusion

			Child Internalizing and Externalizing Problems: Nisonger Child Behavior Rating Form (parent-report)	
Carter-Leno [64]	Quantitative, cross-sectional	Autistic children (n = 257)	*Mental health:* Child Behavior Checklist (teacher-report)<br>*Cognitive flexibility:* Behaviour Inventory Rating of Executive Function (parent-report)	Cognitive flexibility
Charlton [26]	Mixed-methods, cross-sectional	Diagnosed autistic adults (n = 160), Undiagnosed but suspecting adults (n = 139), non-autistic adults (n = 41)	*Autistic traits:* RAADS-14<br>*Stimming, sensory challenges, and wellbeing:* 15-question questionnaire generated for this specific study (self-report)	Stimming, sensory sensitivity
Charlton [91]	Quantitative, cross-sectional	Older autistic adults, ages 40+ (n = 388)	*Quality of life:* World Health Organization Quality of Life measure<br>*Social support:* Duke Social Support Index<br>*Depression:* Patient Health Questionnaire - 9 item version	Inclusion/<br>Social Support
Cook [44]	Systematic review of quantitative studies	Quantitative studies (n = 29)	n/a— systematic review	Autistic camouflaging
Cooper [33]	Quantitative, cross-sectional	Autistic participants ages 16+ (n = 272) and typically-developing controls ages 16+ (n = 267)	*Autism collective self-esteem:* Study-specific measure focusing on how positively autistic individuals viewed autistic people as a group<br>*Individual self-esteem:* Rosenberg Self-Esteem Scale<br>*Depression:* The Trait Depression Scale	Social identity, beliefs about autism
Corden [31]	Mixed-methods, cross-sectional	Autistic adults (n = 151)	*Autistic traits:* Ritvo Autism and Asperger Diagnostic Scale - 14-item version<br>*Self-esteem:* Rosenberg Self-Esteem Scale<br>*Autistic Identity*: Questionnaire on Disability Identity and Opportunity + one study-specific open-ended question	Social identity, beliefs about autism
Crompton [87]	Qualitative, cross-sectional	Autistic adults (n = 12)	*Autistic traits:* Autism Quotient - 50 item version<br>*Social experiences:* No standardized measures— study-specific semi-structured interview	Inclusion

Fernandez-Prieto [78]	Quantitative, cross-sectional	Autistic children and adolescents (n = 79)	*Sensory processing:* Child Sensory Profile (parent-report)<br>*Internalizing and externalizing problems:* Child Behavior Checklist<br>*Executive functioning:* Derived from Child Behavior Checklist	Executive functioning, sensory sensitivity
Greenaway [20]	Quantitative, cross-sectional	Autistic boys (n = 41) and typically-developing boys (n = 42)	*Depression:* Children's Depression Inventory (self- and parent-report)<br>*Cognitive flexibility:* Dysfunctional Attitudes Scale for Children (self-report) and Wisconsin Card Sort Task (researcher-administered)<br>*Perfectionism:* Child and Adolescent Perfectionism Scale (self-report)	Cognitive flexibility, perfectionism
Grove [30]	Quantitative, cross-sectional	Autistic adults (n = 687)	*Special Interests:* Special Interests Motivation Scale<br>*Subjective Wellbeing:* Satisfaction with Life Scale	Special interests
Hedley [15]	Quantitative, cross-sectional	Autistic children, (n = 36)	*Social comparison:* Social Comparison Scale<br>*Depression:* Children's Depression Inventory	Awareness of differences
Hedley [14]	Quantitative, cross-sectional	Autistic individuals, ages 17-55 (n = 71)	*Loneliness:* University of California Los Angeles Loneliness Scale<br>*Depression:* Patient Health Questionnaire - 9 item version	Loneliness
Hollocks [21]	Quantitative, longitudinal	Autistic individuals, 16 at first visit and 23 at second visit (n = 81)	*Autism:* Clinically diagnosed using the Autism Diagnostic Observation Schedule and Autism Diagnostic Interview – Revised<br>*Cognitive Inflexibility:* Opposite Worlds tasks, Trail Making task, Card Sorting task (study-specific, researcher-administered)<br>*Depression:* Beck Depression Inventory (self-report)	Cognitive inflexibility
Hull [46]	Qualitative, cross-sectional	Autistic individuals ages 16+ (n = 92)	*Autism:* Self-report of whether participants had received a clinical diagnosis<br>*Camouflaging:* No standardized measure— study-specific interview questions	Autistic camouflaging
Kapp [27]	Qualitative, cross-sectional	Autistic adults (n = 31)	*Autism:* Self-report of whether participants had received a clinical diagnosis<br>*Stimming:* No standardized measure— study-specific interview questions	Stimming

Lai [47]	Quantitative, cross-sectional	Autistic women (n = 30) and autistic men (n = 30)	*Autism:* Clinical assessment done by research team, Autism Quotient - 50, and Reading the Mind in the Eyes test<br>*Depression:* Beck Depression Inventory<br>*Camouflaging:* Brain activity measured during a study-specific Go/No-Go task	Camouflaging
Lei [63]	Quantitative, cross-sectional	Autistic adolescents (n = 43) and non-autistic adolescents (n = 39)	*Autism:* Clinical diagnoses gathered from medical records or electronically-reported by parent<br>*Camouflaging:* Camouflaging Autistic Traits Questionnaire (self-report)<br>*Depression:* Revised Children's Anxiety and Depression Scale (self-report and parent-report)	Camouflaging
Lei [41]	Systematic review and meta-analysis	Quantitative studies (n = 15)	n/a— systematic review	Cognitive inflexibility
Maitland [32]	Quantitative, cross-sectional	Clinically-diagnosed autistic adults (n = 160) and self-diagnosed autistic adults (n = 24)	*Autism:* Self-reporting of diagnostic status<br>*Social identification:* Social Identification measure, for autism and for other communities participants identified with<br>*Depression:* Beck Depression Inventory	Social identity and belonging
Mazurek [59]	Quantitative, cross-sectional	Clinically-diagnosed autistic adults, ages 18-62 (n = 108)	*Autism*: Autism Quotient - Short (28-item version)<br>*Relationship quality:* Unidimensional Relationship Closeness Scale<br>*Depression:* Patient Health Questionnaire - 9-Item version	Social identity and belonging
Moore [51]	Quantitative, cross-sectional	Autistic adults, ages 18+ (n = 426)	*Autism:* Self-reporting of diagnostic status<br>*Intolerance of Uncertainty:* Intolerance of Uncertainty Scale – short form (self-report)<br>*Depression:* Hospital Anxiety and Depression Scale	Intolerance of uncertainty and sensory sensitivity
Neuhaus [71]	Quantitative, cross-sectional	Autistic children and adolescents (n = 2,079)	*Autism:* Clinically-assessed by clinicians on the research team<br>*Internalizing and Externalizing Problems:* Child Behavior Checklist<br>*Social functioning:* Social Responsiveness Scale	Emotion regulation; social-communication characteristics
Nuske [73]	Quantitative, cross-sectional	Autistic children and adolescents (n = 106)	*Autism:* Clinically-assessed by clinicians on the research team<br>*Loneliness:* Asher Children's Loneliness Scale (self-report)<br>*Self-regulation:* Behavioral Regulation Index (caregiver report)	Loneliness, emotion regulation

			Internalizing and Externalizing Problems: Behavior Assessment Scale for Children, Second Edition (teacher report)	
O'Hagan [92]	Qualitative, cross-sectional	Autistic adolescents (n = 3), parents of those autistic adolescents (n = 3), and teachers (n = 3)	*Autism:* Parents reported previous clinical diagnosis<br>*Inclusion:* Study-specific semi-structured interview	Inclusion
Patel [79]	Quantitative, cross-sectional	Autistic adolescents (n = 25), and non-autistic controls (n = 24)	*Autism:* Clinically-assessed by clinicians on the research team<br>*Anger rumination:* Anger Rumination Scale (self-report)<br>*Depression:* Mood and Feelings Questionnaire— Short Form (self-report)	Emotion regulation
Pfeiffer [93]	Quantitative, cross-sectional	Parents of autistic children and adolescents (n = 50)	*Autism:* Child's clinical diagnosis reported by parent<br>*Sensory sensitivity:* The Sensory Profile, The Adolescent/Adult Sensory Profile (parent-report)<br>*Depression:* Children's Depression Inventory Adapted Parent's Version	Sensory sensitivity
Raymaker [94]	Qualitative, cross-sectional	Autistic adults (n = 10)	*Autism:* Participants self-reported diagnostic status<br>*Autistic burnout:* Assessed through study-specific semi-structured interview questions	Chronic stress, autistic burnout
Reyes [70]	Quantitative, cross-sectional	Autistic children (n = 22) and typically-developing children (n = 22)	*Autism:* Clinically confirmed by clinicians on the study team using the Autism Diagnostic Observation Schedule<br>*Social-communication:* Vineland Adaptive Behavior Scale (parent-report)<br>*Emotion Regulation*: Emotion Regulation Checklist and Emotion Reaction Questionnaire (parent-report)<br>*Internalizing and Externalizing Problems:* Strengths and Difficulties Questionnaire (parent-report)	Social-Communication Characteristics, Emotion Regulation
Rossow [24]	Quantitative, cross-sectional	Autistic pre-schoolers (n = 54)	*Autism:* Clinically confirmed by clinicians on the study team using the Autism Diagnostic Observation Schedule<br>*Sensory sensitivity:* Sensory Processing Scale Inventory, Sensory Assessment of Neurodevelopmental Differences<br>*Depression:* Behavior Assessment System for Children Third Edition-Parent Rating Scale-Preschool	Sensory sensitivity
